# Exploring CSF neurofilament light as a biomarker for MS in clinical practice; a retrospective registry-based study

**DOI:** 10.1177/13524585211039104

**Published:** 2021-08-16

**Authors:** Igal Rosenstein, Markus Axelsson, Lenka Novakova, Kaj Blennow, Henrik Zetterberg, Jan Lycke

**Affiliations:** Department of Clinical Neuroscience, Institute of Neuroscience and Physiology, Sahlgrenska University Hospital, Sahlgrenska Academy, University of Gothenburg, Gothenburg, Sweden; Department of Clinical Neuroscience, Institute of Neuroscience and Physiology, Sahlgrenska University Hospital, Sahlgrenska Academy, University of Gothenburg, Gothenburg, Sweden; Department of Clinical Neuroscience, Institute of Neuroscience and Physiology, Sahlgrenska University Hospital, Sahlgrenska Academy, University of Gothenburg, Gothenburg, Sweden; Department of Psychiatry and Neurochemistry, Institute of Neuroscience and Physiology, Sahlgrenska Academy, University of Gothenburg, Mölndal, Sweden/Clinical Neurochemistry Laboratory, Sahlgrenska University Hospital, Mölndal, Sweden; Department of Psychiatry and Neurochemistry, Institute of Neuroscience and Physiology, Sahlgrenska Academy, University of Gothenburg, Mölndal, Sweden/Clinical Neurochemistry Laboratory, Sahlgrenska University Hospital, Mölndal, Sweden/Department of Neurodegenerative Disease, UCL Queen Square Institute of Neurology, University College London, London, UK/UK Dementia Research Institute, University College London (UCL), London, UK; Department of Clinical Neuroscience, Institute of Neuroscience and Physiology, Sahlgrenska University Hospital, Sahlgrenska Academy, University of Gothenburg, Gothenburg, Sweden

**Keywords:** Multiple sclerosis, cerebrospinal fluid, neurofilament light protein, prognosis, therapy

## Abstract

**Background:**

Neurofilament light (NFL) has been increasingly recognized for prognostic and therapeutic decisions.

**Objective:**

To validate the utility of cerebrospinal fluid NFL (cNFL) as a biomarker in clinical practice of relapsing-remitting multiple sclerosis (RRMS).

**Methods:**

RRMS patients (*n* = 757) who had cNFL analyzed as part of the diagnostic work-up in a single academic multiple sclerosis (MS) center, 2001–2018, were retrospectively identified. cNFL concentrations were determined with two different immunoassays and the ratio of means between them was used for normalization.

**Results:**

RRMS with relapse had 4.4 times higher median cNFL concentration (1134 [interquartile range (IQR) 499–2744] ng/L) than those without relapse (264 [125–537] ng/L, *p* < 0.001) and patients with gadolinium-enhancing lesions had 3.3 times higher median NFL (1414 [606.8–3210] ng/L) than those without (426 [IQR 221–851] ng/L, *p* < 0.001). The sensitivity and specificity of cNFL to detect disease activity was 75% and 98.5%, respectively. High cNFL at MS onset predicted progression to Expanded Disability Status Scale (EDSS) ⩾ 3 (*p* < 0.001, hazard ratios (HR) = 1.89, 95% CI = 1.44–2.65) and conversion to secondary progressive MS (SPMS, *p* = 0.001, HR = 2.5, 95% CI = 1.4–4.2).

**Conclusions:**

cNFL is a robust and reliable biomarker of disease activity, treatment response, and prediction of disability and conversion from RRMS to SPMS. Our data suggest that cNFL should be included in the assessment of patients at MS-onset.

## Introduction

Multiple sclerosis (MS) is an immune-mediated neurodegenerative disease of the central nervous system (CNS). Monitoring of relapsing-remitting MS (RRMS) patients involves relapse rate, disability scoring, and detection of new/enlarging magnetic resonance imaging (MRI) lesions, measures that rely on subjective assessments. There is an unmet need for additional, more objective and quantifiable biomarkers.^1^ Neurofilament light (NFL) is a biomarker of axonal injury that has become the most promising soluble biomarker for assessment of RRMS.^2^ Although accumulated data show that cerebrospinal fluid NFL (cNFL) reflects disease activity^3^ and therapeutic response,^4^ its clinical utility has been limited by the need for repeated lumbar punctures (LPs). The development of ultrasensitive immunoassays enabled determinations of very low NFL concentrations in blood,^5^ making NFL a potential biomarker for clinical practice. Several studies have shown that the associations between cNFL and clinical/MRI measurements^6,7^ are also true for plasma NFL (pNFL).^8,9^ Although pNFL highly correlates with cNFL,^9^ the sensitivity of NFL to detect activity in RRMS seems to be higher in cerebrospinal fluid (CSF).^10^

cNFL has been a certified analysis at Sahlgrenska University Hospital since 2001, which gave us a unique opportunity to validate the clinical utility of cNFL in a real-world setting over an 18-year period. The objective was to confirm cNFL as a biomarker of disease activity, treatment response, and prediction of disability and conversion to secondary progressive MS (SPMS).

## Materials and methods

### Study population

**Figure 1.**
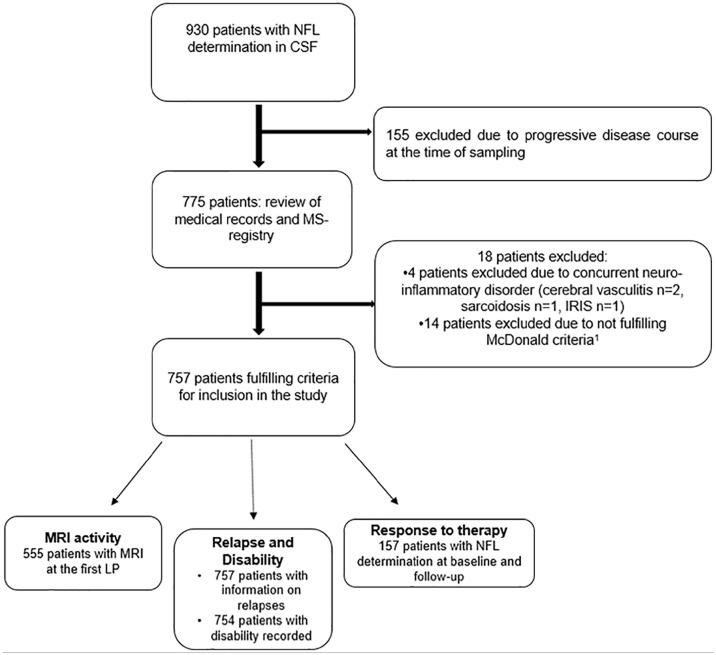
Flow chart of the selection of patients with relapsing-remitting multiple sclerosis fulfilling study criteria. CSF: cerebrospinal fluid; MS: multiple sclerosis; NFL: neurofilament light; IRIS: immune reconstitution inflammatory syndrome.

Three sources were combined to identify eligible patients: the Swedish Multiple Sclerosis Registry (SMSreg, http://www.msreg.net),^11^ archived data of cNFL concentrations analyzed at the Neurochemistry Laboratory, and electronic health record of patients at Sahlgrenska University Hospital. Patients fulfilling the revised McDonald criteria for MS^12^ who had at least one LP between 2001 and 2018, including analysis of cNFL, were retrospectively retrieved (*n* = 930, Figure 1). Excluded from the survey were patients with progressive course at the time of the first LP (*n* = 155). After reviewing patient records, 18 patients were excluded: 14 did not fulfill the diagnostic criteria for MS,^12^ and 4 had another concurrent neuro-inflammatory disorder, in addition to MS making 757 patients eligible for inclusion in the study (Figure 1). Three patients lacked consecutive registrations of disability and were excluded from analysis of progression.

### LPs

A first LP was performed as part of the diagnostic work-up (*n* = 757). A subset of patients had a second LP at follow-up (*n* = 157): 112 for evaluation of treatment response, 12 as follow-up of high NFL baseline levels, 11 to rule out progressive multifocal leukoencephalopathy (PML), and 22 due to suspected relapse.

### Clinical and MRI measurements

The SMSreg contains data on relapse onset and type, disability determined with Expanded Disability Status Scale (EDSS),^13^ and the number of new/enlarging T2- and contrast enhancing lesions on MRI. A relapse was defined as an episode of neurological symptoms lasting 24 hours or longer that could not be explained by another cause^12^ and that occurred within 90 days before the time of baseline sampling. Relapses were classified as optic neuritis (ON), myelitis, infratentorial (IT), supratentorial (ST), and multifocal (MF). Patients were dichotomized into patients with evidence of disease activity (EDA) and those with no evidence of disease activity (NEDA) according to NEDA-3^14^ (no clinical relapses; no confirmed disability worsening (CDW) for 6 months (6-CDW), and no new T1 gadolinium-enhanced lesions/new/newly enlarging T2-lesions).^15^ CDW was defined as an increase in EDSS score with at least 1 point from baseline sustained between two follow-up visits separated in time by no less than 6 months (1.5 point if EDSS at baseline was 0, 0.5 points if the baseline EDSS ⩾ 5.5). Patients not fulfilling NEDA-3 were classified as having active MS/EDA-3. The cohort was also dichotomized into RRMS patients who had EDSS < 3 along the total observational time, including the last visit and those that reached confirmed disability of EDSS ⩾ 3. In addition, patients who remained RRMS at the last visit were compared with those who converted to SPMS. SPMS was defined as steadily increasing objectively documented neurological disability of 1 year or more, independent of relapses.^12^ In patients who did not reach the milestones of EDSS 3 or SPMS, disability was determined with EDSS at the last visit, provided that it was unchanged/not preceded by a recent relapse within the last 6 months. Brain and spinal cord MRI were performed on 1.5 and 3.0T machines, essentially according to Swedish radiological guidelines.^16^ The recorded type and number of MS lesions were according to the review of the neuroradiologist. To associate cNFL levels with MRI disease activity, only MRIs performed 6 weeks before/after LPs were assessed.^17^

### Evaluation of treatment response

Patients who had a subsequent LP were either treatment-naïve (*n* = 43), had initiated a first-line treatment (*n* = 44, interferon-β *n* = 10, glatiramer-acetate *n* = 4, teriflunomide *n* = 7, and dimethyl-fumarate *n* = 23), or switched to a second-line therapy (*n* = 70, natalizumab *n* = 49, fingolimod *n* = 10, rituximab *n* = 5, and alemtuzumab *n* = 6).

### NFL immunoassays

All cNFL analyses were performed by board-certified laboratory technicians in the Clinical Neurochemistry Laboratory at the Sahlgrenska University Hospital, Mölndal. Two different methods for cNFL analyses have been used (2001–2018). The first was an in-house enzyme-linked immunosorbent assay (ELISA) with a lower limit of detection of 250 ng/L,^18^ which was later improved to 125 ng/L.^19^ Over time, both NFL assays have had a coefficient of variation of 15.5%. The second method was a more sensitive sandwich ELISA method (NF-light ELISA kit; UmanDiagnostics AB, Umeå, Sweden) with the lower limit of quantification (LLoQ) of 31 ng/L and with intra-assay and inter-assay coefficients of variation of 10%.^9^ To correct for the differences between these assays, the ratio of means between the second method and its two predecessors was used for normalization. The process of normalization has been reported previously.^20^ Age-adjusted upper limits of the reference range utilized in clinical practice were used to determine whether cNFL levels were elevated or normal. These upper limits are <380 ng/L (<30 years), <560 ng/L (30–39 years), <890 ng/L (40–60 years), and <1850 ng/L (>60 years). These reference values are based on NFL determinations from 120 healthy control subjects without history, symptoms, or signs of neurological or psychiatric disorders, using the upper 95% percentile as the cutoff. They had neither any significant systemic disorder, nor diabetes mellitus or high BMI. Previous or current tobacco smoking was unknown.

### Statistics

Nonparametric tests were used since cNFL levels were nonnormally distributed. Statistical calculations involving whole NFL values were adjusted for age, sex, and disease duration using quantile regression analysis. The Mann–Whitney test was used for comparisons of two groups such as relapse versus no relapse and MRI activity. The Kruskal–Wallis test and false discovery rate test, the two-stage linear step-up procedure of Benjamini, Krieger, and Yekutieli were used for comparison between different relapse types. Correlation of cNFL with the number of contrast-enhancing lesions was calculated with the Spearman-rank correlation coefficient. The receiver operating characteristic (ROC) curve estimations were performed with the assumption of nonparametric distribution. Sensitivity and specificity were calculated using Youden’s index. Kaplan–Meier survival analysis was used to investigate the predictive value of cNFL, where the date of achieving the studied milestones versus the date of the last visit in patients who did not achieve milestones were used for censoring. Patients achieving EDSS milestones at baseline were not excluded. Statistical significance and hazard ratios (HR) were determined by the log-rank test. Wilcoxon matched-pairs signed rank test was used to analyze treatment effects on cNFL concentrations, and statistical significance was determined using two-stage step-up (Benjamini, Krieger, and Yekutieli). Statistical significance was assumed at *p* < 0.05.

### Ethics

All patients included in this study had given consent to be registered in the SMSreg. The study has been approved by the Swedish Ethical Review Agency (Dnr: 2019-01199).

## Results

Demographic and clinical characteristics are presented in Table 1.

**Table 1.** Demographic and clinical characteristics of study population.

Demographic data	Patients (*n* = 757)
Gender, number (%)	
Female	517 (68.3%)
Male	240 (31.7%)
Mean age, years (range)	36.5 (8–74)
Mean follow-up time, years (range)	8 (2–17)
Time from onset to diagnostic LP, months (range)	38.2 (0–473.2)
Disability	Patients (*n* = 754)
Mean baseline EDSS (range)	1.9 (0–8)
Mean EDSS at last visit (range)	2.1 (0–8)
MRI activity	Patients (*n* = 555)
Days between LP and MRI, mean (range)	9.2 (0–42)
MRI brain + spinal cord/brain	296/259
Relapse	Patients (*n* = 757)
Relapse/no relapse	518/239
Type of relapse (%)	
No relapse	31.6
Optic neuritis	13.7
Myelitis	22.6
Infratentorial	13.6
Supratentorial	12.9
Multifocal	5.5
Treatment response	Patients (*n* = 208)
No DMT, *n*	57
First-line DMT	53
Interferon-β	14
Glatiramer acetate	7
Teriflunomide	9
Dimethyl fumarate	23
Second/third-line DMT	86
Natalizumab	61
Fingolimod	13
Rituximab	5
Alemtuzumab	7

EDSS: Expanded Disability Status Scale; MRI: Magnetic resonance imaging; LP: lumbar puncture; DMT: disease modifying therapy.

### NFL and relapses

**Figure 2.**
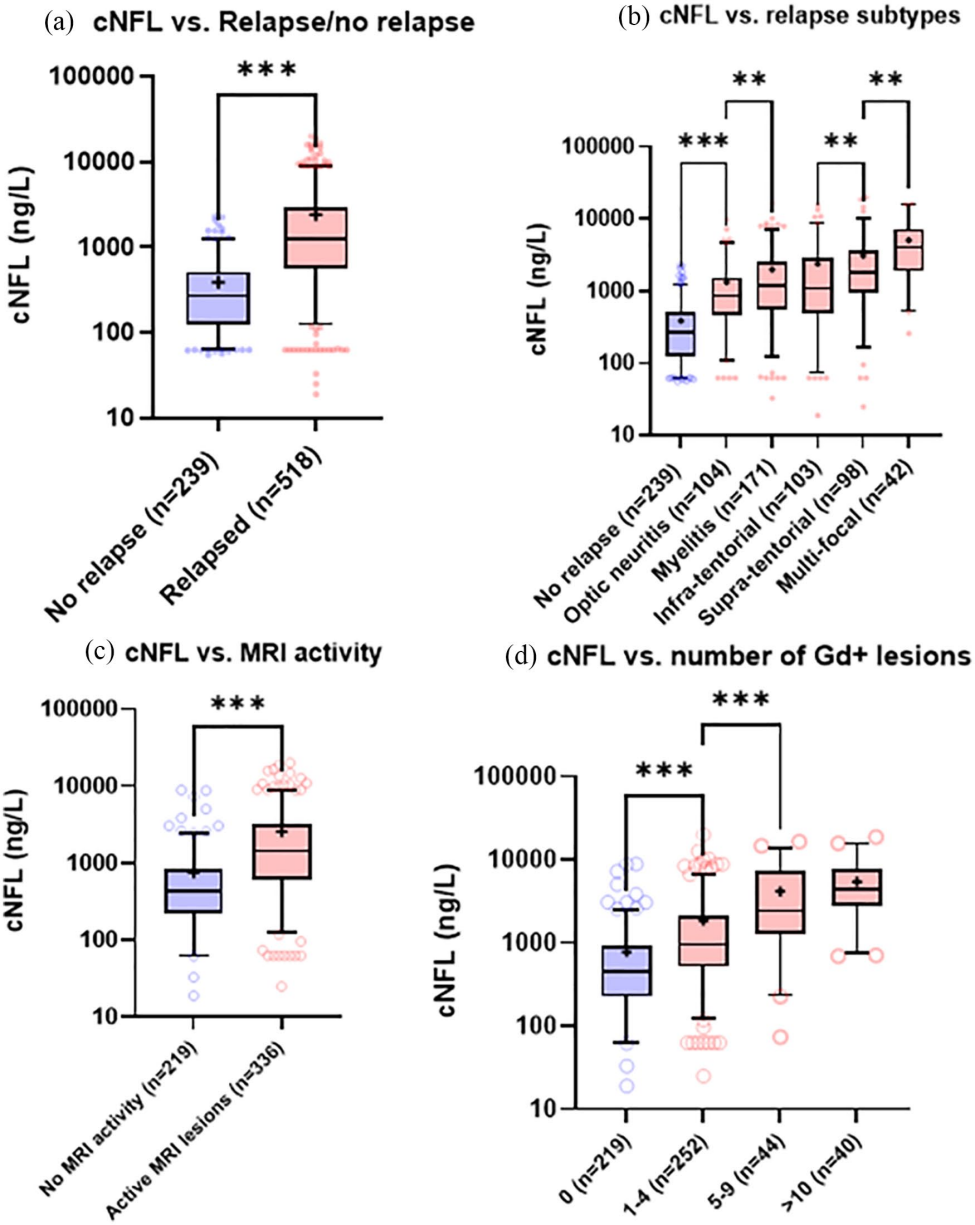
NFL, relapse, and MRI. (a) cNFL levels in patients without concurrent relapse and those who were sampled at the time of a clinical relapse; (b) distribution of cNFL across a spectrum of different relapse-types; (c) cNFL in patients without or without MRI disease activity; (d) cNFL in patients with different amounts of contrast-enhancing lesions on MRI. Box represents IQR. Bar indicates median, whereas + indicates mean. ***p < 0.001; **0.01 ⩽ *p* < 0.05.

Patients with relapse during sampling showed 4.4 times higher median cNFL level (1122 [interquartile range (IQR) 499–2744] ng/L) compared with patients without clinical relapse (264 [IQR 125–537] ng/L, *p* < 0.001, Figure 2(a)). cNFL was higher (*p* < 0.001) in patients with all relapse types when compared with patients without relapse (Figure 2(b)). Median cNFL concentrations were lower in patients with ON (803, [IQR 380–2470] ng/L) compared with myelitis (1117, [IQR 509.5–2436] ng/L, *p* = 0.03) and supratentorial relapses (1601, IQR [495–3272] ng/L, *p* < 0.001), but did not reach statistical significance compared with infratentorial relapses. The highest concentrations were observed in patients with multifocal relapses (4070 [IQR 1908–7281] ng/L, *p* < 0.001). There were no significant differences between myelitis and infratentorial relapses, whereas cNFL levels in supratentorial relapses were significantly higher than both myelitis (*p* = 0.02) and infra-tentorial relapses (*p* = 0.01). There was no significant time-difference from relapse to LP between the different types of relapses. Disease duration and sex did not significantly influence cNFL levels.

### NFL and MRI

cNFL levels were 3.3 times higher (*p* < 0.001) in patients who had contrast-enhancement on MRI (median cNFL 1414 [IQR 606.8–3210] ng/L) compared with patients with no MRI evidence of ongoing disease activity (426 [IQR 221–851] ng/L) and cNFL increased with the number of contrast-enhancing lesions (Spearman’s ρ = 0.523, *p* < 0.001, Figure 2(c) and (d)). Patients with no contrast-enhancing lesions had a median cNFL of 453 ng/L (IQR 224–923), whereas 1–4, 5–9, and >10 Gd+ lesions gave rise to median cNFL of 959 (IQR 513.8–2084) ng/L (*p* < 0.001), 2433 (IQR 1252–7202) ng/L (*p* < 0.001), and 4377 (IQR 2792–7745) ng/L (*p* = 0.03), respectively (Figure 2(d)). Of those patients who did not exhibit Gd+ lesions (*n* = 205), 75 patients did show elevated cNFL (36.6%).

### Sensitivity and specificity of NFL to detect disease activity

**Figure 3.**
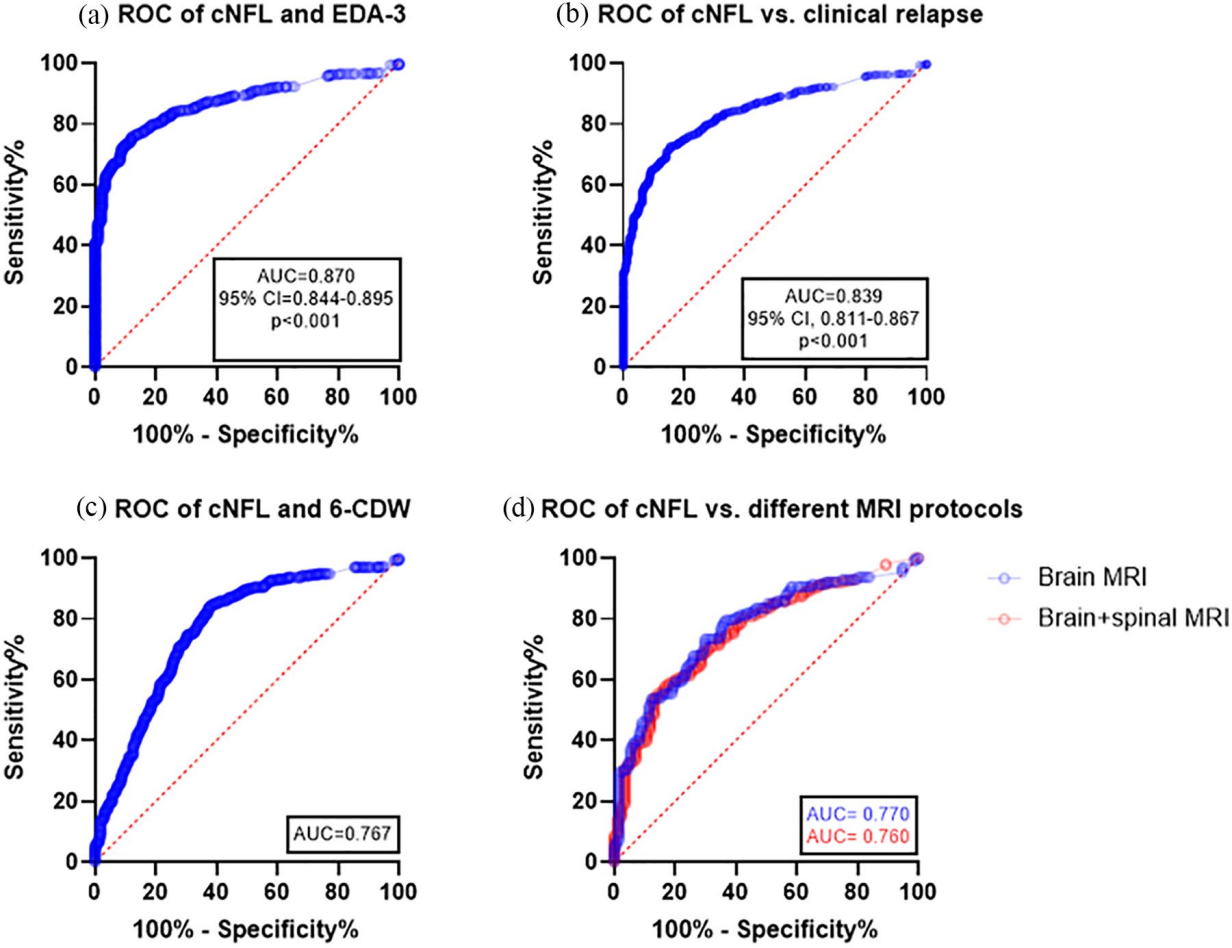
Sensitivity and specificity of NFL to detect disease activity. (a) ROC curves with AUC for CSF NFL indicating sensitivity and specificity to discriminate RRMS patients with EDA-3 and those without concurrent disease activity fulfilling NEDA-3; (b) with or without clinical relapse; (c) with and without CDW; and (d) with or without MRI Gd+ lesions in patients who had only brain MRI versus patients who were surveyed for both brain and spinal cord. ROC: receiver operating characteristic; AUC: area under the curve; cNFL: cerebrospinal fluid neurofilament light; RRMS: relapsing-remitting MS; NEDA: no evidence of disease activity; 6-CDW: 6 month confirmed disability worsening; Gd+: gadolinium-enhancing.

In order to assess the significance of elevated cNFL as a biomarker for disease activity in RRMS, an ROC curve was devised (Figure 3). A cutoff value of 483.5 ng/L gave rise to a sensitivity of 80% (95% CI 76.5–83.1) and specificity of 80% (95% CI 74.2–85.1, Figure 3(a)). A significant proportion of patients with ON (*n* = 36, 35%) had normal levels of cNFL. MRI of the brain and spinal cord were ordered on the discretion of the examining physician based on clinical presentation. The ROC curve was not significantly different between MRI activity obtained from patients examining the brain alone (*n* = 259) and those who examined both the brain and the spinal cord (*n* = 296, Figure 3(d)). MRI of the spinal cord was mostly done when myelitis was suspected (*n* = 148), whereas MRI of the brain alone was done in patients with other clinical presentations (*n* = 189). Another reason for MRI of both brain and spinal cord was suspected RRMS with less obvious origin of clinical symptoms and signs (*n* = 44).

### NFL concentrations and NEDA-3

**Figure 4.**
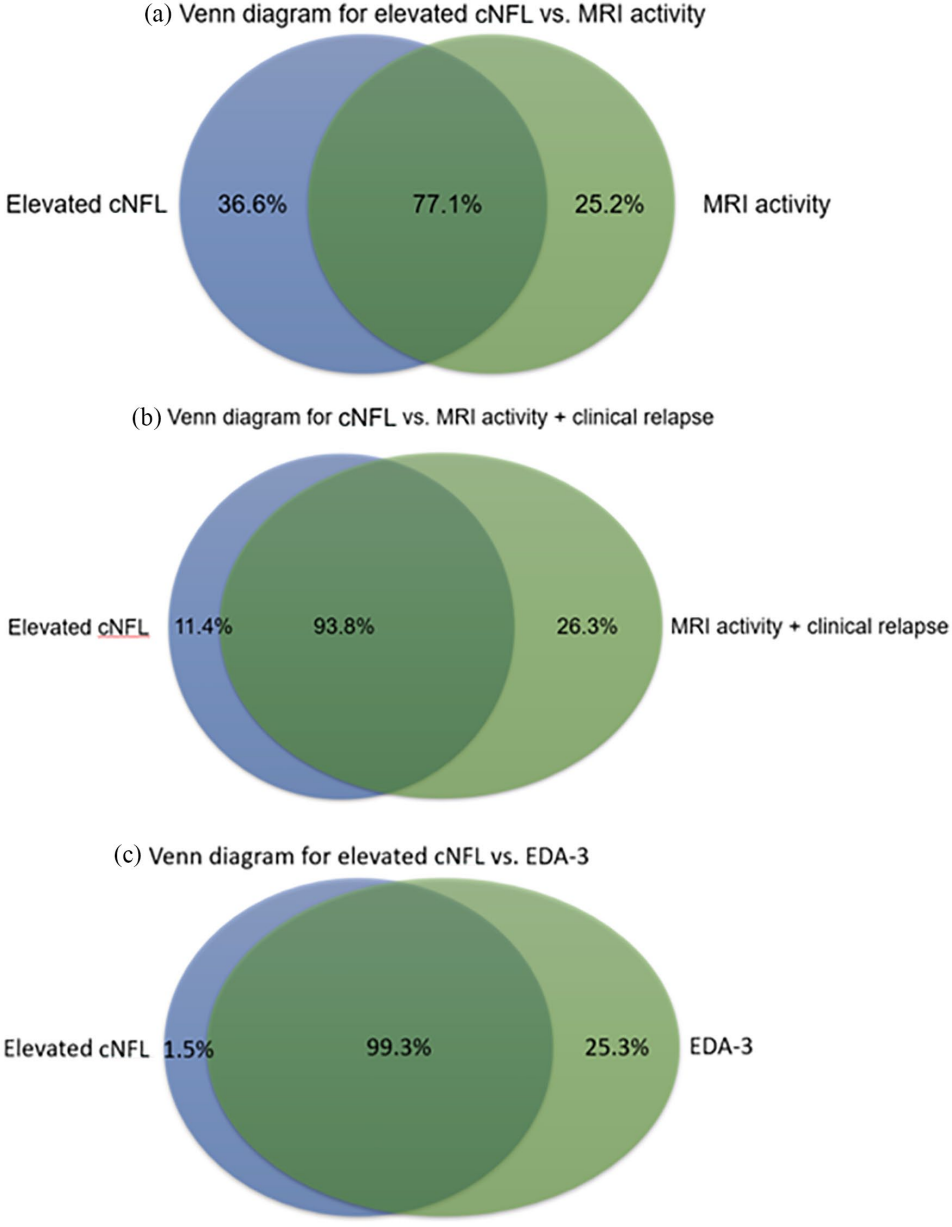
Venn diagrams for elevated cNFL and markers of disease activity. Venn diagrams showing the proportion of patients with (a) elevated cNFL versus MRI activity, (b) elevated cNFL versus MRI activity and clinical relapse, and (c) elevated cNFL versus EDA-3.

Median cNFL at the time of the first LP for the whole cohort (*n* = 757) was 734 (IQR 288.5–1902) ng/L. Patients with active MS or EDA-3 at the time of sampling had five times higher cNFL compared with patients with NEDA-3, that is, stable MS (Table 2). cNFL determinations according to age-specific cutoff values showed an overall sensitivity of 75% and specificity of 98.5% for detection of disease activity, that is, EDA-3. In patients exhibiting MRI activity, MRI activity and relapse, or EDA-3, elevated cNFL was found in 77.1%, 93.8%, and 99.3% of patients, respectively (Figure 4).

**Table 2.** cNFL in active versus stable RRMS patients.

	Stable MS (*n* = 204)	Active MS (*n* = 553)
Gender, female (%)	72.5	66.7
Age, years, mean (range)	40.4 (11–74)	35 (8–70)
cNFL ng/L median (IQR)	234 (125–411.3)	1190 (563.5–2772)
Normal/elevated cNFL	201/3	140/413

MS: multiple sclerosis; cNFL: cerebrospinal fluid neurofilament light; IQR: interquartile range.

### NFL and prediction of disability

**Figure 5.**
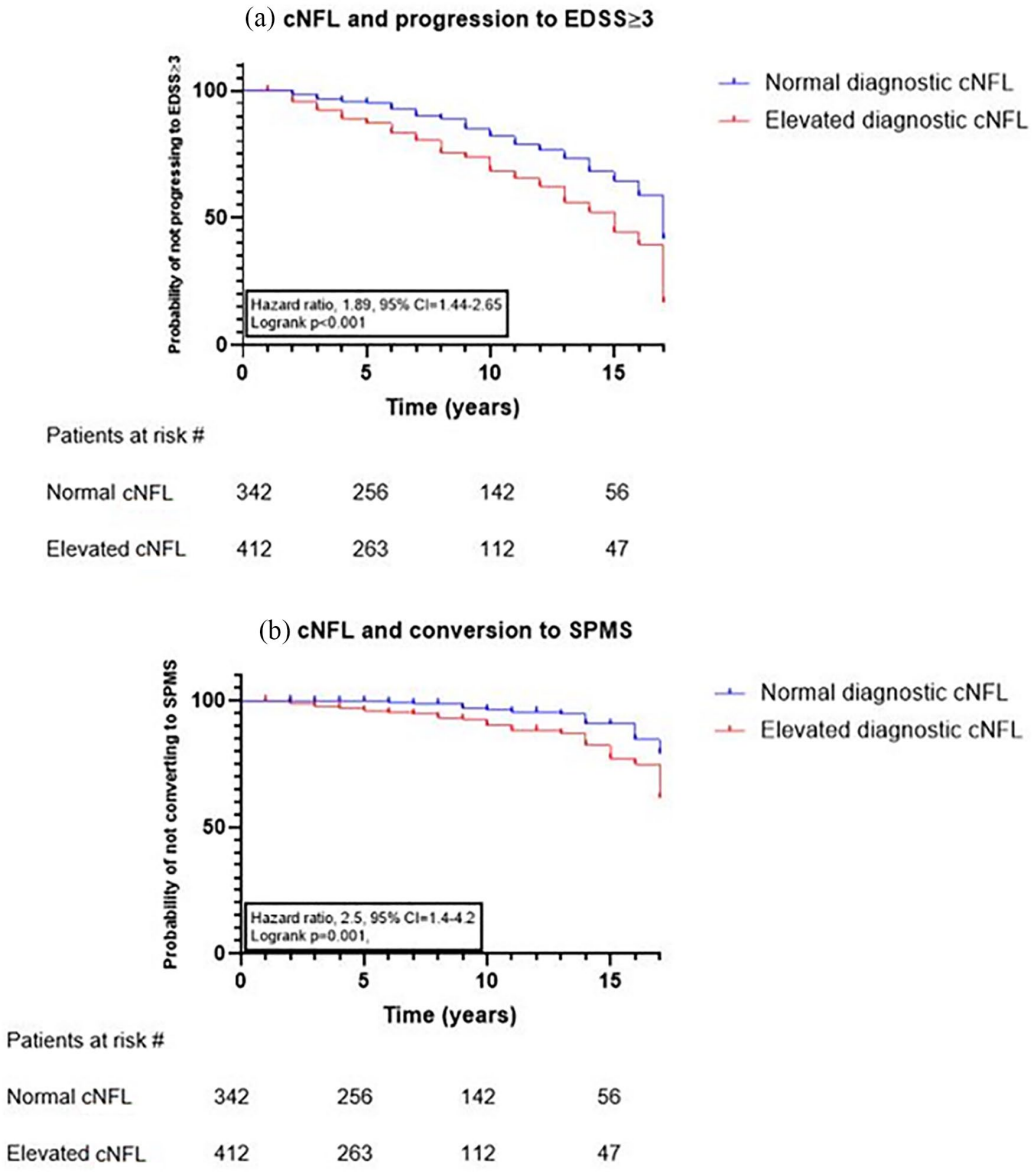
NFL and disability. Kaplan–Meier curves for time to (a) EDSS ⩾ 3 and (b) SPMS in patients with normal versus elevated cNFL levels at diagnosis.

High cNFL (age-dependent cutoff values, high *n* = 412 versus low *n* = 342) at baseline was independently associated with worsening of disability and prediction of progression to EDSS ⩾ 3 (*n* = 205 [27%] *p* < 0.001, HR = 1.89, 95% CI = 1.44–2.65, Figure 5(a)) and conversion to SPMS (*n* = 55 [7%] *p* = 0.001, HR = 2.5, 95% CI = 1.4–4.2, Figure 5(b)). The mean follow-up time for patients who progressed to EDSS ⩾ 3 or SPMS at the time of progression was 9 years (standard deviation [SD] ± 5) and 10 years (SD ± 5), respectively. Mean disease duration was 15 years (SD ± 8) and 18 years (SD ± 9), respectively. Mean age at progression was 49.5 (SD ± 12) and 53 (SD ± 11), respectively. The majority were female (66% and 67%, respectively). At baseline, patients with EDSS < 3, ⩾3, as well as those who progressed to SPMS at follow-up had median cNFL concentrations of 626 (IQR 257–1636) ng/L, 1147 (IQR 495.5–3107 ng/L, *p* < 0.001), and 1717 (IQR 355–4450 ng/L, *p* < 0.001), respectively.

### NFL and treatment response

**Figure 6.**
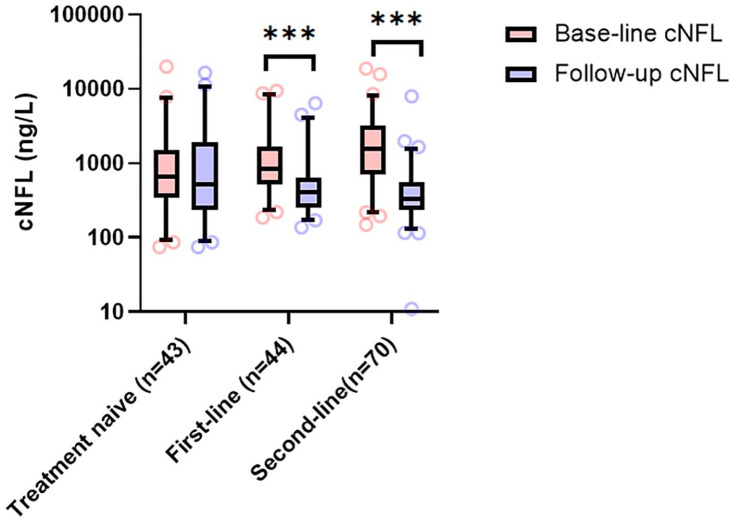
cNFL and treatment response. cNFL levels at baseline and follow-up in patients with RRMS who remained untreated, received a first-line treatment, or switched to a second-line therapy. Median bar indicates median, + indicates mean, and box indicates IQR. ***p *<* 0.001.

Age at baseline and follow-up did not significantly differ between treatment groups. The vast majority of baseline samples were taken during a relapse (83.4%) while most follow-up samples were obtained in a stable phase (80%). The mean interval between LPs was 13.2 months (range 2–26). In treatment-naïve patients who remained untreated at follow-up (*n* = 43), median baseline cNFL was essentially unchanged at follow-up (652 [IQR 346–1527] ng/L versus 523 [IQR 238–1894] ng/L, *p* = 0.91, Figure 6). Follow-up cNFL (406.5 [IQR 250.5–648.5] ng/L) in patients who initiated a first-line therapy (*n* = 44) was significantly lower (*p* < 0.001) compared with baseline (833 [IQR 518.5–1694] ng/L). Patients who switched from a first-line (*n* = 70) to second-line therapy exhibited marked reduction in cNFL (1554 [IQR 697.8–3182] ng/L versus 328.5 [IQR 239.5–545.8] ng/L, *p* < 0.001). Patients who switched to second-line treatment had a significantly higher baseline cNFL than treatment-naïve patients (*p* = 0.001) or those who received first-line therapy (*p* = 0.04). No significant differences in cNFL levels between these treatment groups were observed at follow-up.

## Discussion

We report for the first time real-world data on the utility of cNFL in the clinical practice of RRMS-care. All data were based on cNFL values prospectively obtained since 2001 when cNFL determinations were introduced as a routine analysis at Sahlgrenska University Hospital. We confirmed cNFL as a biomarker in RRMS for disease activity,^3,6,21–23^ treatment response,^4,9,24^ and for prediction of disease severity and clinical course.^25–27^

We show that baseline cNFL is increased across a spectrum of clinical relapses, with the lowest in ON and the highest in multifocal relapses. This ability of cNFL to differentiate between different cNFL levels with different clinical types of relapse has not been described previously. The cNFL concentration most likely reflects the extension of the immune-mediated attack, but may also depend on the location of new lesions. NFL concentrations correlate with both the number and volume of lesions in MS,^28^ and a similar association was shown with lesion volume in acute ischemic stroke.^29^ Although the vicinity of lesions to the subarachnoid space may also influence cNFL concentrations, we found no significant differences in cNFL concentrations between relapses of other origin than ON. Our data do not support that distance between the lesion and the site of LP is of importance for cNFL concentration.

We could also confirm that NFL levels are higher in patients with MRI activity and that cNFL rises with the increasing number of contrast-enhancing lesions.^22–24^ cNFL had high sensitivity but a lower specificity for MRI activity. The reduced specificity compared with EDA-3 seemed to not be due to missing activity in the spinal cord. In a separate analysis, the ROC curve did not differ significantly between patients who performed MRI of the brain alone and those who had both brain and spinal cord examined. MRI scans were ordered on the basis of clinical signs and symptoms, and suspicion of myelitis was usually confirmed on spinal cord scans. However, some patients had MRI of the spinal cord due to relapses of less obvious origin or as a diagnostic procedure.^30^ Our findings show that MRI scans based on clinical presentation and suspicion of lesion origin had relatively high precision and the sensitivity and specificity of cNFL to detect MRI activity was similar in patients, including MRI of the spinal cord as in those with MRI of the brain alone.

We confirmed that high NFL levels are good predictors of patients not fulfilling NEDA-3.^10^ In line with the results of a previous study,^31^ a proportion of patients not showing MRI activity and/or fulfilling NEDA-3 criteria still had elevated NFL levels, which indicate that NFL determination may complement other measures of disease activity. We found, however, that a significant proportion of patients with ON had normal cNFL levels at diagnosis and no evidence of ongoing disease activity on MRI. This had a major impact on the sensitivity of NFL to support NEDA-3. The sensitivity and specificity of high cNFL concentrations was higher for EDA-3 than for clinical relapses or MRI activity alone, supporting that including CDW-6 gives rise to a more accurate representation of the pathological process that causes axonal injury in MS.

In 2011, we showed for the first time that the cNFL concentration was markedly reduced after natalizumab treatment in active MS.^24^ Thereafter, several studies have shown similar reduction or even normalization of NFL after DMT in both CSF and blood.^3,4,9^ In our real-world material, we confirm the utility of cNFL as a biomarker for monitoring treatment effect in clinical practice. Our data reflect the degree of DMT efficacy and the effect of switching treatment to more effective DMTs. Determination of pNFL has become an established outcome measure in several trials.^32^ Thus, so far pNFL has been studied mostly at the group level but it is still unclear how it can be used and interpreted for guiding individual clinical decisions. pNFL shows high interindividual variability, age dependency, and there is impact from other confounding factors.^9,33^ Perhaps the most important contribution of our study is that we confirm the predictive value of NFL.^21,25^ cNFL could predict the risk of reaching meaningful milestones as EDSS ⩾ 3 and conversion to SPMS. Several studies have explored pNFL’s ability to predict disease worsening,^34,35^ but cNFL seems to be more precise in individual cases.^10^ Further work is needed to establish the clinical utility of pNFL in prediction of disability worsening and the degree of disease activity in direct comparison with cNFL. Since the presence of CSF oligoclonal IgG-bands was re-incorporated into the revised 2017 McDonald diagnostic criteria as a possibility to fulfill dissemination in time,^12^ most diagnostic investigations include LPs. In addition, there is evidence that NFL is a particularly stable and robust biomarker that does not require special handling and may survive days of transport.^36^

Important limitations in our study is its retrospective design, which may introduce selection bias. Although this may concern cNFL determinations for treatment response, it is less likely for evaluation of its predictive value, since determination of cNFL has been incorporated in the diagnostic work-up and lab-routine at Sahlgrenska University Hospital for over two decades. Over the years, numerous neurologists and radiologists have contributed to the assessment of patients and reviewing MRI. The high interrater variability for EDSS is well known,^37^ and the increased risk associated with multiple assessors probably also concerns classifying relapses, as well as the assessment of MRI lesions. Nevertheless, despite these methodological caveats, we could confirm the utility of cNFL.

In conclusion, our data underline NFL as a sensitive biomarker of disease activity, its usefulness for prediction of disability and clinical course, and for monitoring the DMT response. Our results suggest that NFL determination could be included in clinical practice as a prognostic tool as well as for treatment decisions together with clinical and MRI measures.
